# The Treatment of Nephritis

**Published:** 1903-11-28

**Authors:** 


### THE TREATMENT OF NEPHRITIS.

In discussing some recent suggestions bearing on the treatment of the various forms of nephritis, Dr. Horder[1] remarks that although drugs are of comparatively small value, yet there are few diseases in which treatment, based upon a sound knowledge of the patient's condition and experience of the effects of therapeutics in other cases of the malady, has a greater influence on the course of the disorder. There is too much a tendency, he thinks, to regard illnesses in which drugs have little or no influence as being to a large degree outside the sphere of active help on the part of the doctor. Much good is likely to be achieved in the future by a fuller study of metabolism in cases of nephritis, especially in patients who are in a hospital where accurate clinical research is not attended with insurmountable difficulties. It is by working on these lines that advance in therapeutic knowledge is to be expected. Dr. Horder proceeds to indicate some points which especially need investigation of this kind. The chief of these is the effect which different dietaries have upon the course of the disease. He quotes the results which Professor von Noorden has arrived at in regard to the elimination of food principles in acute nephritis. This author classes the following among the substances excreted with difficulty: urea, creatinin, pigments, hippuric acid, phosphates, inorganic sulphates. The substances easily excreted are; uric acid, xanthin-bases, aromatic substances, ammonia, amido acids, chlorides and carbonates. As regards water, the important observation is made that in the early stages of acute nephritis water is eliminated with great difficulty; later on this difficulty passes away.

As a result of these observations von Noorden lays down the following dietetic principles for patients with acute nephritis.

I. To be avoided:—(1) Because of creatinin produced: meat extracts, meat broths, eggs; (2) because of pigments produced: foods containing hæmoglobin. (3) Because of hippuric acid produced: green vegetables.

II. To be given sparingly:—(1) Because of urea and sulphates produced: milk; (2) because of irritant effect during excretion: water.

III. To be given with impunity: cream, butter, white vegetable soups, puddings made from oatmeal, flour or maize, white bread.

It will be noticed that the usual exclusive milk diet in acute nephritis is discountenanced and the amount of fluids is limited.

For severe cases in which there is partial or complete suppression of urine with increasing œdema and threatened uræmia, von Noorden allows only a pint of milk a day, and avoids other fluids.

Another matter relating to diet in nephritis is the possible harmfulness of sodium chloride. Dr. Horder mentions the recent work done by MM. Widal and Lemierre, which seems to establish a relation between the amount of salt ingested and the œdema in cases of tubular nephritis. The absorption of 10 grammes of salt each day in two patients suffering from this affection led to considerable œdema. In four cases of interstitial nephritis no such effect occurred. Further observations have been made in this direction, and although it is too early to make a positive statement on the matter, it does seem that salt has a tendency to increase œdema and albuminuria in cases of tubular nephritis.

With regard to the treatment of chronic interstitial nephritis it is pointed out that the dietetic principles differ considerably from those to be followed in cases of acute nephritis. The kidney must be spared as much as possible, but the nutrition of the whole organism must be maintained, and especially must the heart muscle be spared and strengthened. Alcohol, pepper, and other condiments, celery, radishes and asparagas must be avoided, so also must the following drugs: salicylic acid, mercury, boric acid, and tar preparations. Coffee, tea, and tobacco are injurious because of their weakening action on the heart muscle. Red meat is not more injurious than white meat, and neither need be limited. Only small quantities of water should be taken, as large quantities of water absorbed from the intestine can only be excreted by means of a high general blood pressure.

[1] Practitioner, November, 1903.

